# Supplementary material for: The Yeast Permease Agp2 Senses Cycloheximide and Undergoes Degradation That Requires the Small Protein Brp1-Cellular Fate of Agp2 in Response to Cycloheximide
Source: Int J Mol Sci. 2023 Apr 10;24(8):6975. doi: 10.3390/ijms24086975 (PMC10138708; doi:10.3390/ijms24086975)
Supplement: Supplementary file 1 [file ijms-24-06975-s001.zip › ijms-2289508-supplementary.pdf]

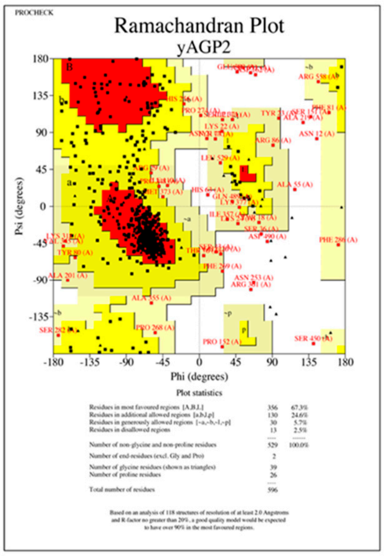

Figure S1. Model validation using PROCHECK. The Ramachandran plot showing that 67.3% of the residues are in the favorable region, 24.6% of the residues in the additionally allowed region and 5.7% of the residues in the generously allowed region. In total 97.5% of the residues are in the allowed region and this indicates a good stereochemical quality of the predicted Agp2 model.

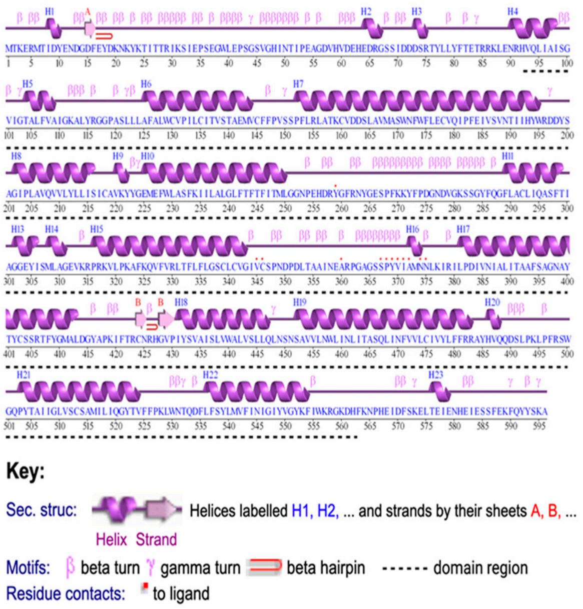

Figure S2. Secondary Structure representation of the predicted Model using PDBsum webserver. The legend describes the different secondary structure elements and domain region present in the predicted model.

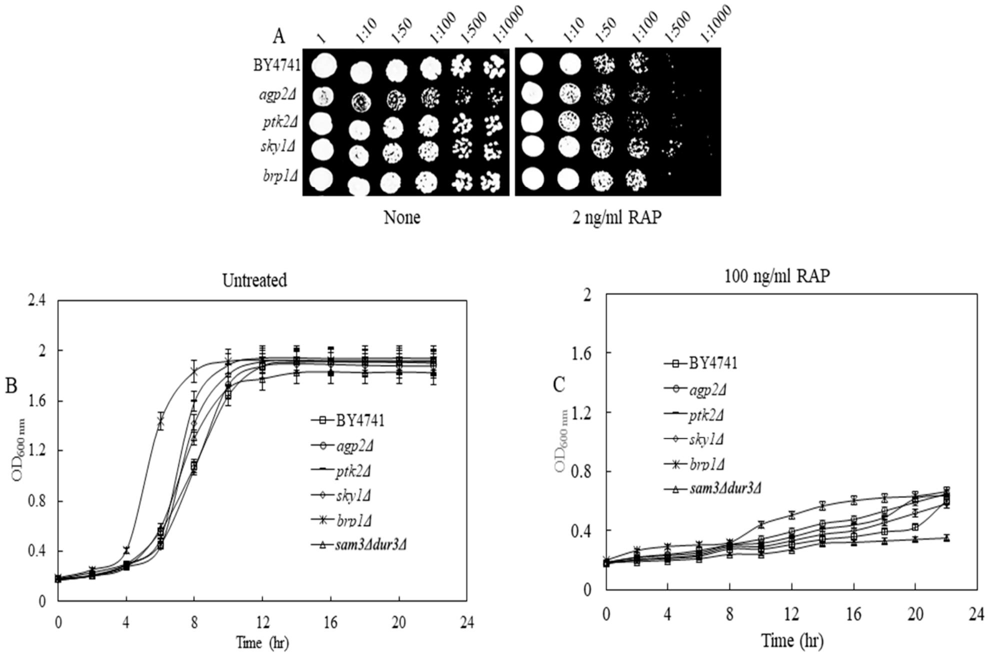

Figure S3. Sensitivity analysis of the indicated strains towards rapamycin. A, Spot test analysis. The experiment was performed as in Figure 4, except the cells were spotted onto solid YPD plates without and with rapamycin (RAP). Plates were photographed after 72 hours of incubation at 30 ºC. B, Growth rate of the indicated cells without and with RAP and monitored as in Figure 4.

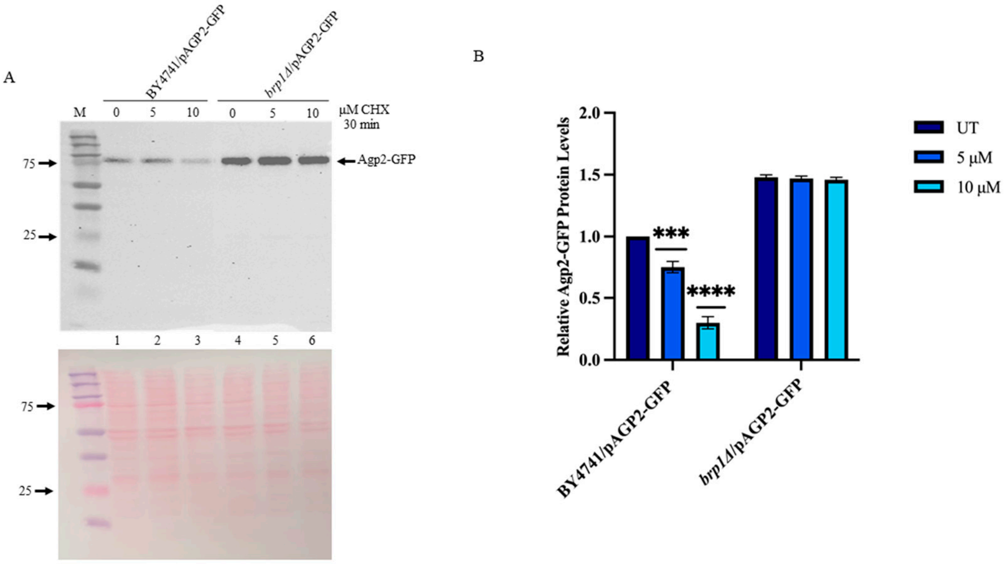

Figure S4. Immunoblot showing that Agp2-GFP is disappearing in the WT but not in the *brp1Δ* mutant upon exposure to CHX. A) Immunoblot analysis of cells treated with increasing concentrations of CHX. The experiment was done as indicated in Figure 5. Ponceau staining of the immunoblot to monitor for protein loading. B) Quantification of the disappearance of Agp2-GFP relative to the untreated sample (lane 1). *** Is equivalent to P-value < 0.001, **** Is equivalent to P-value < 0.0001.

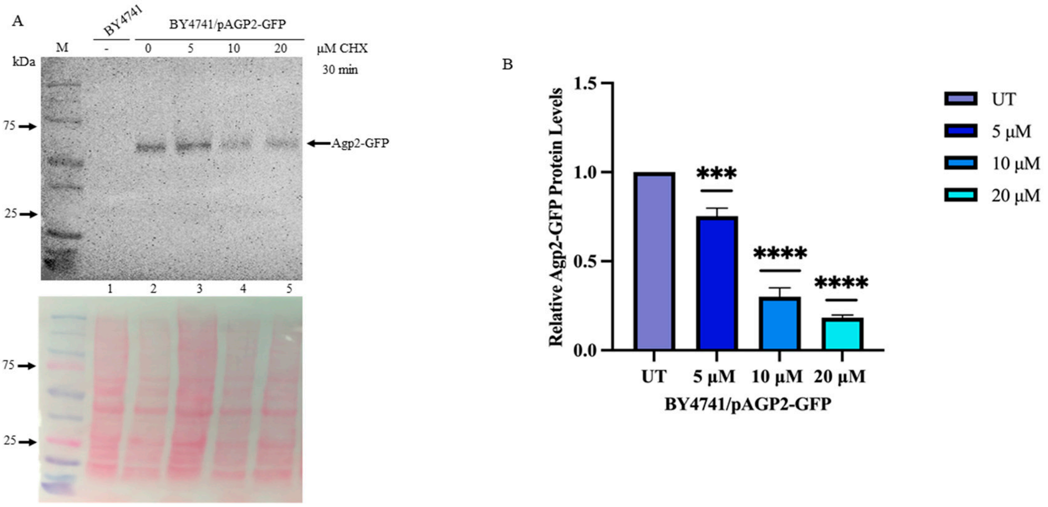

Figure S5. Independent immunoblot analysis of WT cells expressing Agp2-GFP and treated with increasing concentrations of CHX. The experiment was done as indicated in Figure 5. Lane 1, WT cells carrying an empty vector. Ponceau staining of the immunoblot to monitor for protein loading. B) Quantification of the disappearance of Agp2-GFP relative to the untreated sample (lane 2). *** Is equivalent to P-value < 0.001, **** Is equivalent to P-value < 0.0001.

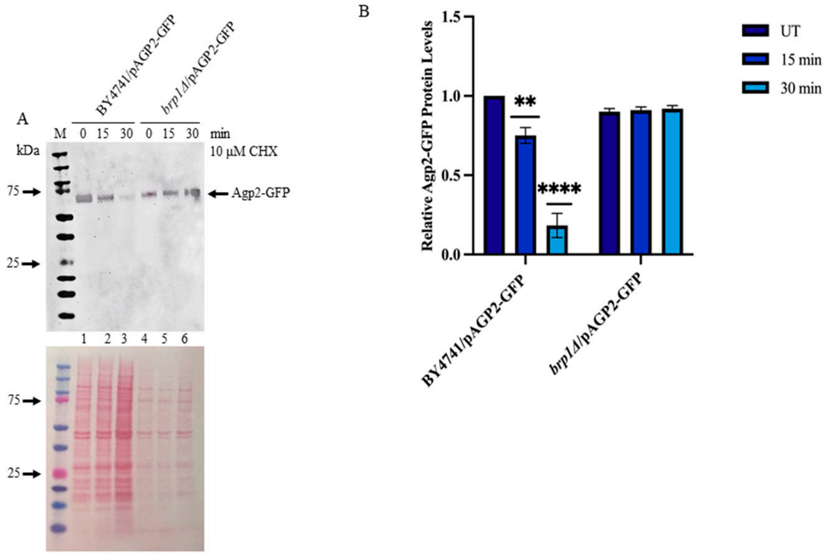

Figure S6. Independent immunoblot analysis showing that Agp2-GFP is disappearing in the WT but not in the *brp1Δ* mutant upon exposure to 10µM CHX for the indicated time points. A) Immunoblot analysis of cells treated with CHX for 0, 15 and 30 mins. The experiment was done as indicated in Figure 5. Ponceau staining of the immunoblot to monitor for protein loading. B) Quantification of the disappearance of Agp2-GFP in the WT and the *brp1Δ* mutant relative to the untreated sample (lane 1 and lane 4, respectively). ** Is equivalent to P-value < 0.01, **** Is equivalent to P-value < 0.0001.

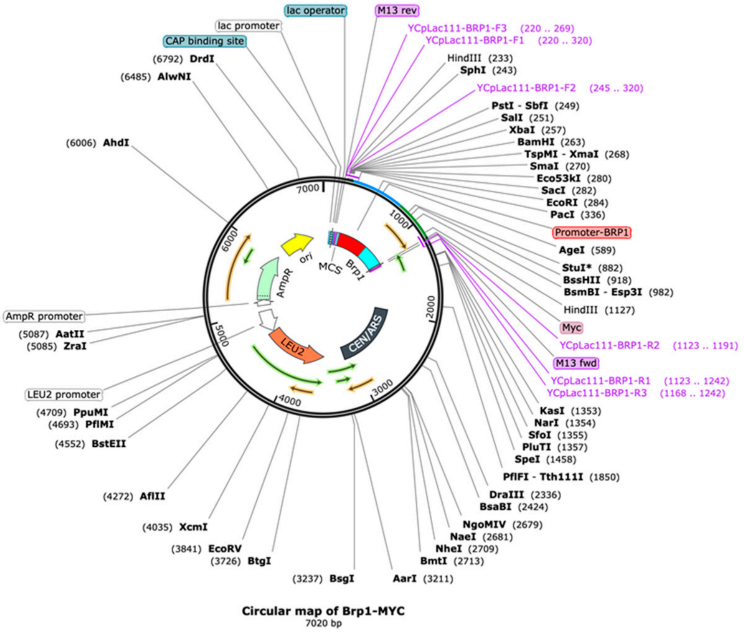

Figure S7. Schematic representation of the circular map of BRP1-MYC construct. The entire BRP1 gene (promoter and coding sequences) was placed in frame with the MYC sequence in the single copy vector YcPlac22 to produce the plasmid pBRP1-MYC. The plasmid map was created using SnapGene.
